# Protective Effects of Neutral Lipids from *Phaeodactylum tricornutum* on Palmitate-Induced Lipid Accumulation in HepG2 Cells: An In Vitro Model of Non-Alcoholic Fatty Liver Disease

**DOI:** 10.3390/molecules31020323

**Published:** 2026-01-17

**Authors:** Marion Peyras, Rose-Marie Orhant, Giuliana Parisi, Cecilia Faraloni, Graziella Chini Zittelli, Vincent Blanckaert, Virginie Mimouni

**Affiliations:** 1 Biology of Organisms, Stress, Health and Environment, Département Génie Biologique, Institut Universitaire de Technologie, Le Mans Université, IUML-FR 3473 CNRS, F-53020 Laval CEDEX 9, France; peyrasmarion@orange.fr (M.P.); rose-marie.orhant@univ.lemans.fr (R.-M.O.); vincent.blanckaert@univ-lemans.fr (V.B.); 2 Department of Agriculture Food Environment and Forestry, University of Florence, Via delle Cascine 5, I-50144 Florence, Italy; giuliana.parisi@unifi.it; 3 Institute for BioEconomy, Department of Biology, Agriculture and Food Sciences, National Research Council, Sesto Fiorentino, I-50019 Florence, Italy; cecilia.faraloni@cnr.it (C.F.); graziella.chinizittelli@cnr.it (G.C.Z.)

**Keywords:** HepG2, metabolic syndrome, obesity, NAFLD, *Phaeodactylum tricornutum*, neutral lipids, polar lipids

## Abstract

Non-alcoholic fatty liver disease (NAFLD), often associated with obesity, has become a serious public health matter. NAFLD is characterized by an excessive lipid accumulation in hepatocytes, mainly stored as triglycerides. The marine microalga *Phaeodactylum tricornutum* is well known for its richness of bioactive compounds, particularly lipids. Therefore, different natural lipid extracts from *P. tricornutum* are deciphered to jugulate or prevent obesity leading to NAFLD. In this study, the main focus was on the effects of purified neutral and polar lipid extracts from *P. tricornutum* in a cellular model of NAFLD. Human HepG2 cells were used and exposed for 24 h to 250 μM palmitate to induce NAFLD with or without microalgal lipid extracts. Data showed that neutral lipid extract presented lower viability and cytotoxic activities on HepG2 at 75 µg/mL. The impact on apoptosis was around 5% and below the threshold. Nevertheless, the use of neutral lipid at 50 µg/mL induced a decrease in the number and size of lipid droplets, and so, preventing NAFLD. On the contrary, the polar lipid extract had no effect on the accumulation of triglycerides in HepG2 cells. To conclude, neutral lipid extract seemed to be a good candidate to prevent NAFLD.

## 1. Introduction

Obesity is considered to be a multifactorial chronic disease characterized by an excessive fat accumulation due mainly to changes in lifestyle, such as diets rich in sugars and saturated fats, increased sedentary behaviors, and short time sleeping [1].

Data from the Global Health Observatory (GBO) published in 2016 by the World Health Organization (WHO) indicate that 13.1% of the global population was obese and 38.9% was overweight, while the data in 1975 reported only 4.7% and 21.5%, respectively. Furthermore, in 2016, 15.1% of women and 11.1% of men were obese, while 39.2% of women and 38.5% of men were overweight [2]. By 2025, global obesity prevalence is predicted to reach 18% in men and exceed 21% in women [3]. Obesity leads to many complications such as type 2 diabetes and causes insulin resistance, increased risk of high blood pressure and atherosclerosis, and liver diseases such as non-alcoholic fatty liver disease (NAFLD) [4]. Obesity can thus lead to an accumulation of fats in the liver (steatosis) which can be associated with inflammation of the liver (steatohepatitis) [5]. NAFLD is a silent disease that can progress, if left untreated, and lead to severe forms such as inflammation of the liver, characterized by the presence of scar tissue, resulting in liver fibrosis that can lead to cirrhosis or liver cancer [6].

NAFLD frequently affects people with obesity which is a major public health issue because of its high prevalence in Western countries. Animal studies and in vitro tests are currently being developed for investigating the components of natural product medicines [7]. The main manifestation of NAFLD is hepatic lipid accumulation in the form of lipid droplets (LDs), known as hepatic steatosis (fatty liver) [8]. Current treatments for NAFLD generally aim to reduce triglyceride (TG) accumulation, often using thiazolidinediones (TZDs) and fibrates, which are known to lower TG levels in hyperlipidemia, diabetes, and metabolic syndrome [7]. Alternatively, natural medicines for the treatment of NAFLD have a long and successful history of controlling disease without prominent side effects [7]. However, active compounds in natural medicine responsible for lowering hepatic TG levels are yet poorly characterized. For instance, in our previous study using total lipophilic extracts (TLE) and carotenoid extracts (CE) of *P. tricornutum*, it has been shown that both TLE and CE prevented the increases in triglycerides, total cholesterol, and cholesterol esters levels in palmitate-treated HepG2 cells [9]. In the same study, the total lipophilic extract also decreased the mRNA expression levels of genes involved in lipogenesis (ACACA, FASN, SCD, and DGAT1) and cholesterol esterification (ACAT1/SOAT1). In addition, the total lipophilic extract also downregulated the LXR/NR1H3 and SREBF1 genes, which are involved in lipogenesis regulation [9]. Interesting results were also obtained in vivo by using Wistar rats fed by a high fat diet with or without *P. tricornutum* as food supplementation. Data highlighted the beneficial effects of this marine microalga in reducing the metabolic disorders, notably hyperlipidemia, associated with metabolic syndrome that could induce NAFLD [10].

In this context, the aim of this study was to determine, after separating and purifying the neutral lipids (NL) and polar lipids (PL) fractions from total lipophilic extract, which lipid fraction, NL and/or PL fractions, have the most effective beneficial effect against NAFLD. First of all, viability and cytotoxicity tests have been performed on purified NL and PL extracts from the microalga *P. tricornutum* in palmitate-treated HepG2 cells. Then, we have investigated the effects of the purified NL and PL extracts on hepatic steatosis in HepG2 cells. The objective was to determine which concentrations of NL and PL extracts from *P. tricornutum* was slightly or non-toxic for HepG2 cells. This, to be able to identify the most effective lipid extract for reducing lipid droplets (LDs) in HepG2 cells, by reducing LD size, number, and triglyceride (TG) content in LDs.

## 2. Results

### 2.1. Fatty Acid (FA) Composition of Neutral (NL) and Polar Lipid (PL) Extracts from P. tricornutum

By comparison of the composition of neutral and lipid extracts (Table 1) it appears that n-3 long chain polyunsaturated fatty acids (PUFAn-3) are extremely well represented in NL with an amount of 1003.83 while it reaches only 229.83 mg/100 g in PL. To emphasize PUFAn-3, the main difference has been observed with eicosapentaenoic acid (EPA; C20:5n-3) with amounts of 922.61 and 179.33 mg/100 g in NL and PL, respectively. Regarding the value in percentage, C22:6n-3 (DHA) resulted 1.62% of the total fatty acids in the neutral fraction and 3.32% in the polar one. Concerning PUFAn-6, almost 2-fold higher levels were observed in the NL compared to PL (113.00 versus 58.33 mg/100 g, respectively). A relevant difference has been observed for arachidonic acid (ARA, 20:4n6) with an amount of 51.78 in NL versus 18.00 mg/100 g in PL. In addition, PUFAn-4 were preferentially represented in the NL with amounts nearly threefold higher than those observed in PL (310.11 vs. 113.00 mg/100 g). Monounsaturated fatty acids (MUFA) were also associated with the NL fraction, exhibiting amounts of 451.33 mg/100 g compared with 139.33 mg/100 g in the PL fraction.

Finally, saturated fatty acids (SFA) showed higher amounts in the NL fraction (382.28 mg/100 g) than in the PL fraction (285.50 mg/100 g). When the incidence is analyzed, the SFA percentage was markedly higher in the polar fraction (34.50 versus 16.86% of the total fatty acids).

### 2.2. Effect of Neutral and Polar Lipids from P. tricornutum on HepG2 Cell Viability

**Figure 1:**
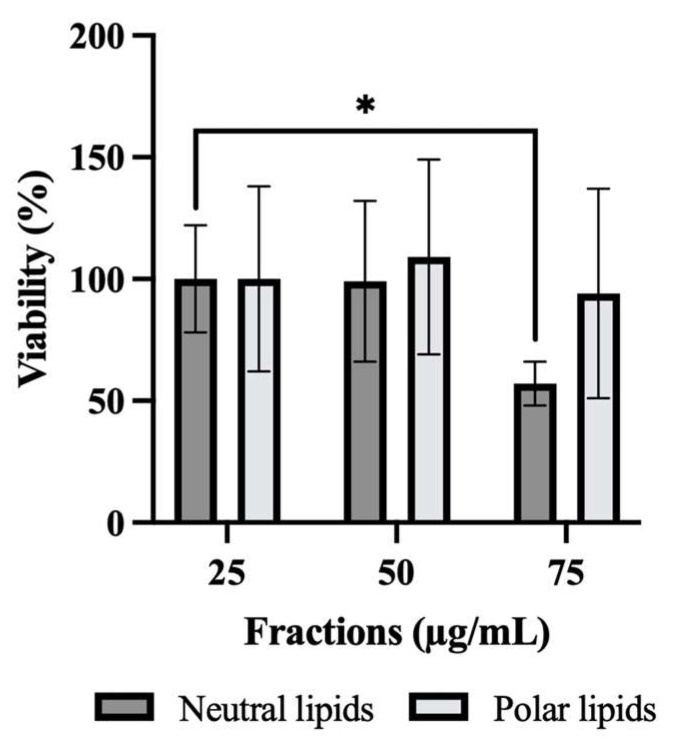
Effect of neutral and polar lipids on HepG2 viability. An MTT method was used. The number of independent experiments was n = 4 and each experiment was performed in triplicate. Statistic was made using ANOVA with a Dunnett test as post hoc. * *p* ≤ 0.05.

Figure 1 shows the viability in percentage as a function of the different concentrations of lipid extracts (25, 50, 75 μg/mL) obtained by the MTT method with an absorbance at a wavelenght of 540 nm. Whatever the concentrations, no difference was observed for PL while a decreased proliferation (*p* ≤ 0.05) was observed with NL at a concentration of 75 µg/mL (*p* ≤ 0.05). These results show that only NL seem to impact the viability of HepG2 cells. The lipid extracts of the microalgae were added to the cells 24 h later in a starving buffer and the absence of fetal calf serum (FCS) led to a cell proliferation arrest in the G0/G1 phase of the cell cycle due to the lack of growth factor [11]. The formazan crystals obtained can be used to determine cell viability. A decrease in percentage would indicate a cytotoxic effect of the lipid extracts on the cells. PL therefore had no cytotoxic effect on the cells whereas NL might have been cytotoxic at a concentration of 75 µg/mL.

### 2.3. Effect of Neutral and Polar Lipids on HepG2 Cell Cytotoxicity

**Figure 2:**
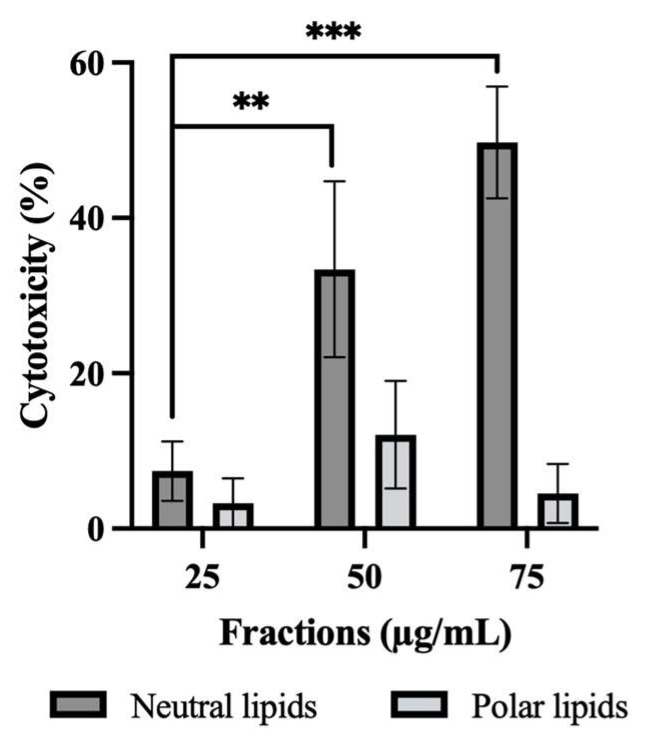
Cytotoxicity of HepG2 cells with neutral and polar lipids. An LDH assay on the cell line was performed. The number of independent experiments was N = 3 and each experiment was performed in triplicate. Statistic was made using ANOVA with a Dunnett test as post hoc. ** *p* ≤ 0.01. *** *p* ≤ 0.001.

Figure 2 shows the percentage of cytotoxicity obtained with the lactate deshydrogenase (LDH) method as a function of the different concentrations of lipid extracts (25, 50, 75 μg/mL). The graph shows that there is no cytotoxicity of PL on HepG2 cells. For NL, a cytotoxicity with an increase in lipid concentration was observed. The LDH enzyme is released during two cellular mechanisms: necrosis and apoptosis [12,13]. The release of this enzyme is very strong during necrosis and decreases during the process while at the level of programmed cell death LDH is observed only at the end of apoptosis. These two mechanisms are differentiable according to the appearance of the cell nucleus. During necrosis, the plasma membrane is altered and LDH is released, allowing the incorporation of propidium iodide (PI) into the nucleus. During apoptosis, the plasma membrane is not damaged. However, the Hoechst reagent allowed a stain of all nuclei making the visualization of condensed nuclei or apoptotic bodies characteristic of the end of apoptosis.

### 2.4. Effect of Neutral Lipids from P. tricornutum on HepG2 Apoptosis

**Figure 3:**
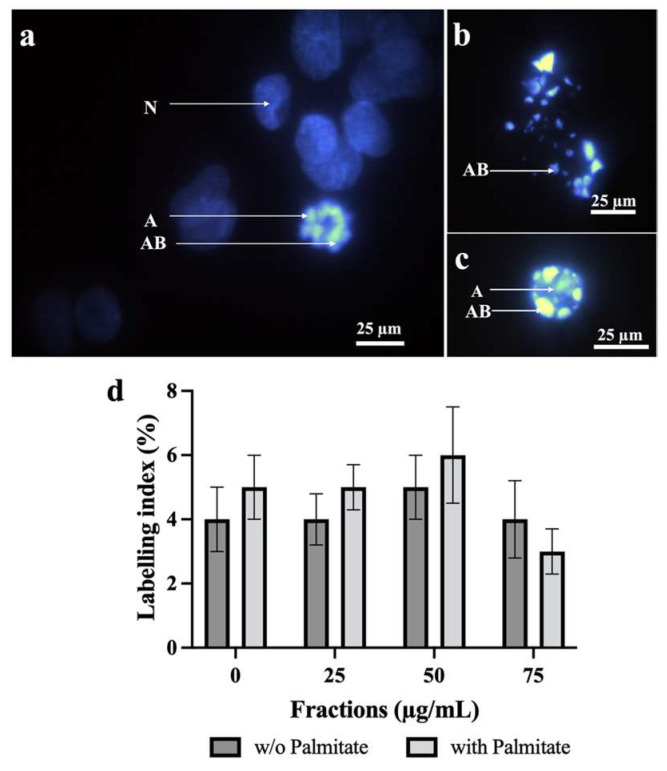
Effect of neutral lipid on HepG2 apoptosis after Hoechst staining of the nuclei. (**a**) Normal nuclei (N) with an apoptotic nucleus (A) composed by apoptotic bodies (AB) were observed; (**b**,**c**) show a late and an early apoptosis, respectively; (**d**) labeling index was obtained by dividing the number of apoptotic nuclei on the total number of nuclei time 100 after or not incorporation of different amounts of neutral lipids. The number of independent experiments was N = 3 and each experiment was performed in duplicate. Statistic was made using ANOVA with a Dunnett test as post hoc. Bar = 25 µm.

Figure 3 shows normal and apoptosis nuclei with or without palmitate on HepG2 cells treated or not with NL. (a) The microphotography presented normal nuclei (N) and an intermediate apoptosis nucleus (A) with the presence of apoptotic bodies (AB); (b) the figure displayed a nucleus in late apoptosis, and (c) a poorly advanced apoptosis presented an early stage close to the condensed nucleus considered as part of anomalous nuclei. The graphic presented in (d) shows no differences between palmitate-treated cells (gray) or without (black) among the different concentrations. The labeling index (LI), in % of the different neutral lipid concentrations, was not statistically different and close to the control (CTRL). As no significant differences on apoptosis LI was observed, it seemed that cytotoxicity was probably due to necrosis of the cells by the NL fraction.

### 2.5. Effect of NL and PL at a Concentration of 50 µg/mL (NL50 and PL50) on Lipid Droplet Size in HepG2 Cells

**Figure 4:**
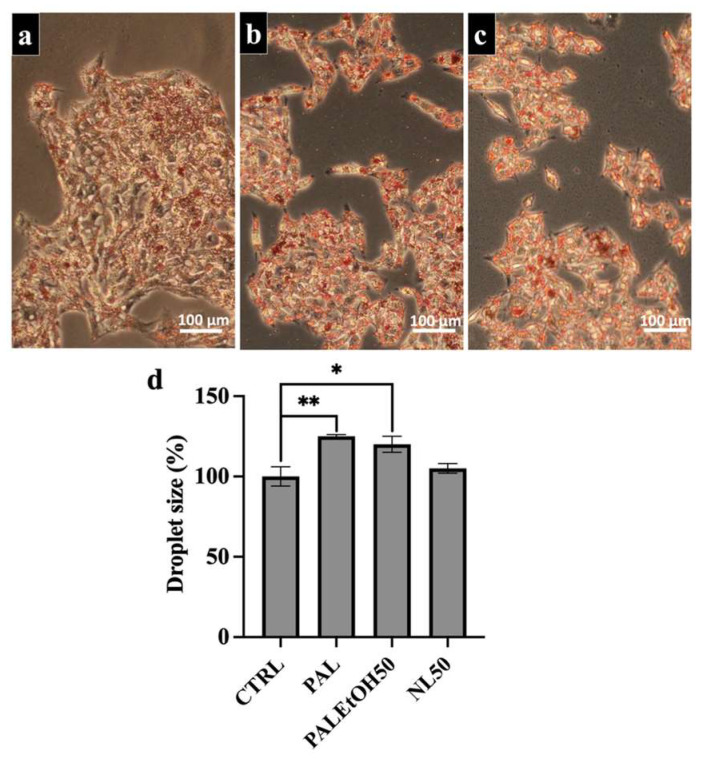
Effect of neutral lipid at 50 µg/mL on HepG2 NAFLD. (**a**) The palmitic acid (PAL)-treated HepG2 cell mimicked the NAFLD; (**b**) PAL + Ethanol Control LN50 (LNEth50); (**c**) PAL + neutral lipid at 50 µg/mL (LN50) and (**d**) average size of the droplets according to ImageJ of HepG2 NAFLD with or without 50 µg/mL of neutral lipids. The number of independent experiments was N = 3 and each experiment was performed in triplicate. Six micrographs per condition were taken and submitted to ImageJ analysis. Statistics were made using ANOVA with a Dunnett test as post hoc. * *p* ≤ 0.05 and ** *p* ≤ 0.01. Bar corresponds to 100 µm.

**Figure 5:**
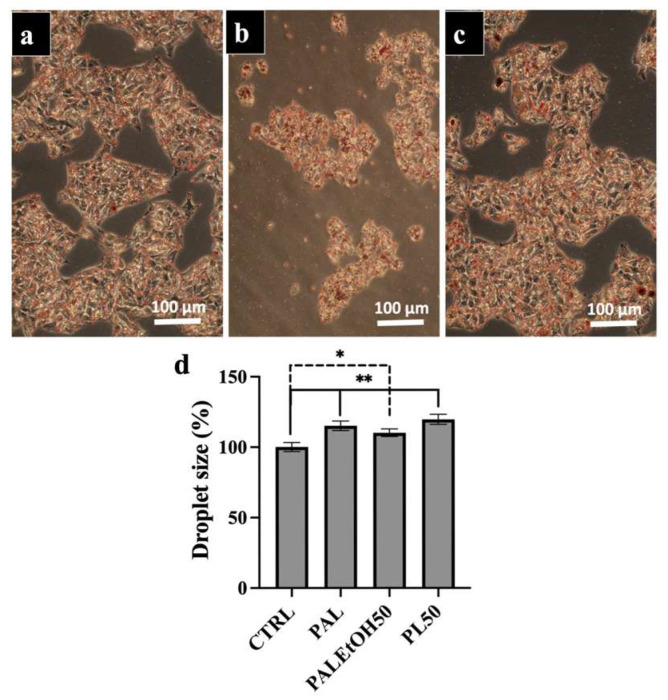
Effect of polar lipid at 50 µg/mL on HepG2 NAFLD. (**a**) The palmitic acid (PAL)-treated HepG2 cell mimicked the NAFLD; (**b**) PAL + Ethanol Control LP50 (LPEth50); (**c**) PAL + polar lipid at 50 µg/mL (PL50) and (**d**) average size of the droplets according to ImageJ of HepG2 NAFLD with or without 50 µg/mL of polar lipids. The number of independent experiments was N = 3 and each experiment was performed in triplicate. Six micrographs per condition were taken and submitted to ImageJ analysis. Statistics were made using ANOVA with a Dunnett test as post hoc. * *p* ≤ 0.05 and ** *p* ≤ 0.01. Bar corresponds to 100 µm.

Figure 4 and Figure 5 show the effects of neutral lipids and polar lipids at 50 µg/mL (NL50 and PL50) on lipid droplet (LD) size in subconfluent HepG2 cells. Cells were treated with (a) palmitate (PAL), (b) a volume of ethanol equivalent to the lipid fraction (PAL+EtOH50), and (c) the lipid fraction at 50 µg/mL (PAL+NL50 or PAL+PL50). LDs were detected by Oil Red O staining. Treatment with NL50 reduced LD size compared to treatment only with palmitate stimulating NAFLD, while treatment with PL50 slightly increased LD size compared to cells treated only with palmitate. Figure 4 and Figure 5d confirm these effects, showing the average LD size measured via ImageJ version 1.53 analysis for NL50 and PL50 treatments, respectively. Ethanol alone did not affect LD size, which remained comparable to PAL-treated cells. Statistical analysis revealed significant differences in LD size between PAL or ethanol treatments and the control (CTRL), with *p* ≤ 0.01 and *p* ≤ 0.05, respectively. No significant difference in average LD size was observed between CTRL and NL50-treated cells, indicating that NL50 was beneficial to restore LD size to normal. In contrast, a significant difference between CTRL and PL50-treated cells indicates that PL50 was unable to restore LD size.

### 2.6. Effect of NL or PL Fractions on Lipid Droplet Accumulation

**Figure 6:**
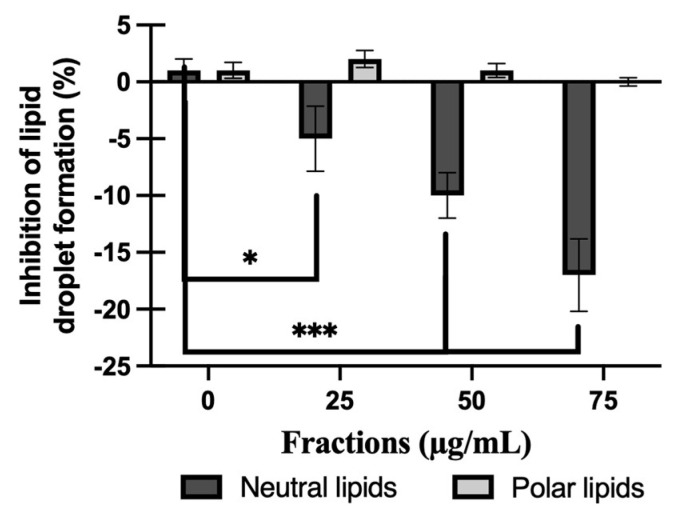
Effect of neutral or polar lipids on NAFLD. Results were obtained by subtraction of absorbances of the respective ethanol fraction and the lipids. The number of independent experiments was N = 3 and each experiment was performed in triplicate. Statistic was made using ANOVA with a Dunnett test as post hoc. * *p* ≤ 0.05 and *** *p* ≤ 0.001.

NL but not PL extracts reduced LD amounts in PAL-treated HepG2 cells, as shown in Figure 6. This result clearly showed that the NL extracts were able to statistically decrease the amount of lipids in the lipidic droplets, with *p* value of 0.001. Thus, the comparison between the size of the LD (Figure 4) and amount at 50 µg/mL (Figure 6) is well in range as a decreased size corresponds to a lower amount of lipid present in the lipidic droplets.

## 3. Discussion

The aim of this study was to investigate the impact of NL and PL fractions from *P. tricornutum* in a cellular model of NAFLD induced by PAL treatment. Twenty-four hours of PAL treatment at a concentration of 250 µM has been used to induce NAFLD without altering cell viability in HepG2 cells [9,14]. To assess the potential protective effects of microalga extracts on PAL-induced NAFLD, NL, and PL extracts must be used at non-toxic concentrations.

NAFLD is a continuum of chronic liver diseases ranging from benign hepatosteatosis to non-alcoholic steatohepatitis (NASH), cirrhosis and primary hepatocellular cancer (HCC). Because of its strong association with the obesity epidemic, NAFLD is rapidly becoming a major public health concern worldwide. This focus is based on recent studies indicating that NASH patients and preclinical mouse models of NASH have low levels of hepatic C20–22 n-3 PUFA. This decline in hepatic PUFA may account for the major phenotypic features associated with NAFLD, including steatosis, inflammation, and fibrosis [15]. In this study, results evidenced that PL had no effects in NAFLD while NL induced a decrease on the accumulation of triglycerides in HepG2 cells. It has also been shown that NL extract presents high cytotoxic effects at 75 µg/mL, medium and mild at 50 and 25 µg/mL, respectively. The 50 µg/mL was considered having the best balance between a cytotoxic effect and the positive effect on the disease. In addition, LDH assay and Hoechst staining of NL showed that cytotoxicity was probably due to necrosis rather than apoptosis; the labeling index was equivalent to the control whatever the concentration of NL used for apoptotic study. It has been shown that NL, leading to micro- and macro-vesicular steatosis and balloon, require increased intracellular lipids leading to apoptosis and/or necrosis [16]. Fatty acid mixtures induce a similar magnitude of fat accumulation and different experimental models of a cellular steatosis profile have been described. On the one hand, the mixture containing the lowest proportion of palmitic acid (oleate/palmitate, 2:1 proportion) was associated with minor toxic and apoptotic effects, thus representing a cellular model of steatosis in which unsaturated fatty acids played a protective role against lipotoxicity, mimicking benign chronic steatosis. On the other hand, a high proportion of PAL (oleate/palmitate, 0:3 proportion) might represent a cellular model of steatosis in which saturated free fatty acids promote an acute harmful effect of fat overaccumulation in the liver [17]. Steatosis, one of the NAFLD states, is common in obese patients [18]. The assay employed here to determine anti-steatosis activity is based on the important role of the liver in fatty acid metabolism and used HepG2 cell lines exposed to PAL, which is known to induce steatosis in cells [9,16].

Research on lipid accumulation in tissues is a key feature of many metabolic diseases. Thus, techniques for imaging and quantifying lipids in various tissues are important for understanding and evaluating the overall metabolic status. A protocol that detects NL and LD morphology by oil red O (ORO) staining of HepG2 cells has been performed in this study. To do so, microphotographs of PAL-treated cells with and without NL or PL fractions were used followed by ImageJ processes enabling the average size of the lipid droplets present in cells. It has been shown that a procedure using ORO staining on fixed tissue sections described here is well suited for the comparison of hepatic lipid accumulation in diet-induced and genetically induced diabetic mice [19]. The beneficial effect on lipid droplet accumulation was observed only with NL extract, as shown in Figure 6. However, the PL fraction might also have an effect in other pathologies such as anti-inflammatory and anti-thrombotic activities [20]. The relevant difference in EPA content between the NL and PL of *P. tricornutum* (922.611 versus 179.333 mg/100 g microalga) could be responsible for the different behavior observed. In a previous study, it has been shown that total lipophilic extract from *P. tricornutum* has preventive effects against NAFLD by reducing the accumulation of hepatic lipid levels [9]. Moreover, potential anti-obesity, anti-steatosis, and anti-inflammatory properties of crude microalgae extracts from the microalgae *Chlorella vulgaris* and *Chlorococcum amblystomatis* under different growth conditions were also found [21]. Using a zebrafish bioassay, it has been shown by fluorescent image analysis that lipid accumulation associated with steatosis has been described as having decreased in NAFLD, due to the catalyze allowing the formation of monounsaturated fatty acids (MUFAs) [22]. A high number of FA (n. 38) was found in the NL fraction but those present in a percentage greater than 1% were only 14 in total. As previously found, NL are the major lipid fraction (2267 versus 827 mg/100 g microalga) of *P. tricornutum* total lipids, with palmitic (8.89%), palmitoleic (14.22%) acids and, mainly, EPA (40.69%) in the higher percentages. Richness in EPA also characterizes the PL fraction of the algae, in which it accounts for 21.67% of the total FA, 22 of which were found to be less than 1%. Although the percentage of DHA was much lower than that of EPA, in both lipid fractions, the presence of these FA makes this microalga very interesting from a health point of view and, consequently, a candidate ingredient for natural functional foods. Even though different culture processes and conditions produce differences in lipid classes and FA composition in microalgae, *P. tricornutum* is widely known for its significant amounts of PUFA, mainly n-3 PUFA and, in particular, EPA.

With regard to the fatty acid profiles of the NL and PL extracts from *P. tricornutum*, it appeared that eicosapentaenoic acid (EPA) and MUFA were the most representative FA in neutral lipids compared to PL. The high level of EPA in NL extract can explain the protective effect on NAFLD. Indeed, it is well established that EPA improves hepatic metabolism and reduces inflammation in HepG2 cells [23,24].

Several studies have reported that n-3 PUFAs, in particular EPA and docosahexaenoic acid (DHA), reduce the progression of NAFLD by lowering hepatic levels of TG and cholesterol esters [25]. These beneficial effects could be explained by the ability of the n-3 PUFA to control the activity and/or expression of transcription factors that regulate the expression of genes encoding proteins involved in de novo lipogenesis, fatty acid oxidation, fat uptake from the circulation and its assimilation into lipids, and very low density lipoprotein assembly and secretion [14]. n-3 PUFAs exert preventive effects against dyslipidemia but also insulin resistance, inflammation, and oxidative stress, all involved in the establishment of NAFLD. In addition, a decrease in the FA n-3/n-6 ratio has been found to favor a pro-inflammatory state and leads to adverse metabolic health outcomes [26,27] including hepatic steatosis [28]. Other studies suggest that MUFA intake exert beneficial effects on NAFLD. Human studies evidenced that MUFA decreased liver fat [29], whereas cell studies found that MUFA induced hepatic steatosis [30]. In addition, it has been shown that antioxidant peptides from monkfish swim bladders is able to ameliorate NAFLD in vitro by suppressing lipid accumulation and oxidative stress via regulating the AMPK/Nrf2 pathway [31]. Of course, these results are preliminary and there are many other assays that take into account liver damages and can be used to show the effects of these extracts on NAFLD, and in particular, that of NL. These assays can be due to the search for and use of markers such as ALT, AST, TC, TG, and inflammation factors levels, such as Mean Platelet Volume, or serum markers such as CK-18 [32].

The use of in vitro platforms offering 3D models, or chips type could be considered in order to better understand how NAFLD can be controlled or is working [33].

## 4. Materials and Methods

### 4.1. Materials

#### 4.1.1. Human Hepatocyte Model

The hepatocyte model chosen is the HepG2 cell line obtained from the American Type Culture Collection (ATCC). This immortalized cancer cell line derived from a human hepatocarcinoma, developed from the cells of a liver epithelium from a 15-year-old male Caucasian teenager.

Due to a high potential for morphological and functional differentiation in vitro, HepG2 cells are used as an in vitro model for investigating hepatic steatosis and NAFLD-related disturbances that occur in the early stages of NAFLD. As an immortalized cell line, HepG2 offers genetic stability, reproducibility, low variability, and ease of culture, enabling standardized studies of hepatic steatosis mechanisms [34].

#### 4.1.2. *P. tricornutum* Lipid Extracts

The NL and PL extracts were supplied by the CNR-IBE laboratory (Florence, Italy) and were obtained from the marine microalga *P. tricornutum.* The *P. tricornutum* biomass was produced in closed photobioreactors (500–1000 L working volume) installed under a greenhouse during the late spring. The cultures were grown in artificial seawater enriched with F medium nutrients [35] and harvested at the early stationary phase. At harvesting time, cultures were centrifuged to obtain a paste of about 80% moisture that was then frozen, lyophilized, and powdered. The powdered biomasses were mixed to obtain a single batch and stored at −20 °C until using.

Total lipids from *P. tricornutum* were extracted then purified into two groups: (i) NL consisting mainly of TG forming a carbon reserve and playing a role as an energy reserve, and (ii) PL represented mainly by phospholipids and glycolipids which are the main components of the microalgal membranes and participate in their stability.

### 4.2. Methods

#### 4.2.1. Cell Culture of HepG2

Cells previously frozen in liquid nitrogen at −196 °C were thawed at room temperature. They were transferred to a Falcon tube containing 10 mL of EMEM (Eagle’s Minimal Essential Medium, ATCC) culture medium supplemented with 0.4% penicillin/streptomycin (PAN Biotech, Aidenbach, Germany) and 15% fetal calf serum (ATCC) and compose the complete EMEM medium. After centrifugation at 500 rpm for 10 min at 4 °C, the cell pellet was resuspended in 6 mL of complete EMEM medium. The cell suspension obtained was seeded in a well of a 6-well plate and then incubated in the incubator at 37 °C and 5% CO_2_. The maintenance, cell amplification, and various cell treatments were carried out under aseptic conditions under a laminar flow hood. The HepG2 cells seeded at a rate of 1000 cells/cm^2^ then form an adherent cell that develops into monolayers and aggregates. To maintain cells or for the first step of assays, after removal of the complete EMEM medium, the cells fixed in the flasks were rinsed with 5 mL of saline phosphate buffer (PBS, Phosphate-Buffered Saline, pH 7.4) for T75 and 2.5 mL for T25 flasks. Once PBS was removed, 1 mL of sterile trypsin-EDTA (PAN Biotech) was dispensed for T25 vials and 2 mL for T75 flasks and incubated for 10 min at 37 °C. The addition of trypsin-EDTA followed by an incubation of 10 min at 37 °C detached the cells. The action of trypsin was inhibited by adding the complete EMEM. Using a Malassez cell, the cell concentration was determined to take the desired quantity of cells. This volume is then introduced into a new flask containing 6 mL of complete culture medium for a T25 and 12 mL for a T75. The incubations were performed in the incubator at 37 °C and 5% CO_2_. When the confluence reaches 70–80%, cell passages were carried out.

#### 4.2.2. NL and PL Extracts from *P. tricornutum*

The NL and PL extracts from the microalga *P. tricornutum* were obtained from the CNR-IBE laboratory (Florence, Italy). *P. tricornutum* total lipids were obtained according to Folch et al. [36], then they were gravimetrically quantified. Lipid fraction (NL and PL) separation was obtained with the method described by Juaneda and Rocquelin [37]. To obtain the fractions (NL and PL) from the total lipid extract, a solid phase extraction was conducted with a Sep-Pack Silica column (Waters, Milford, MA, USA) by first injecting methanol into the column to recover the PL fraction and then chloroforme to recover the neutral lipid fraction. Then, solvent was evaporated, and the lipid fractions were both gravimetrically quantified and utilized for the analysis of their overall FA profile.

The FAs were determined in NL and PL extract after trans-esterification to methyl esters (FAME), using a base-catalyzed trans-esterification [38]. The FA composition was determined by gas chromatography (GC) using a Varian GC 430 gas chromatograph (Agilent, Palo Alto, CA, USA), equipped with a flame ionization detector (FID); a Supelco Omegawax™ 320 capillary column (30 m × 0.32 mm i.d., 0.25 μm film and polyethylene glycol bonded phase; Supelco, Bellefonte, PA, USA) was utilized. The oven temperature was held at 100 °C for 2 min, increased to 160 °C over 4 min at the rate of 12 °C min^−1^, and then increased to 220 °C over 14 min, at the rate of 3 °C min^−1^, and kept at 220 °C for 25 min. The injector and the detector temperatures were set at 220 °C and 300 °C, respectively. A quantity of 1 μL of sample in hexane was injected into the column with the carrier gas (helium) kept at a constant flow of 1.5 mL min^−1^. The split ratio was 1:20. Chromatograms were recorded with the Galaxie Chromatography Data System 1.9.302.952 (Varian Inc., Palo Alto, CA, USA). FAs were identified by comparing the FAME retention time with those of the Supelco 37 component FAME mix standard (Supelco, Bellefonte, PA, USA) and quantified through calibration curves, using tricosanoïc acid (C23:0) (Supelco, Bellefonte, PA, USA), as internal standard.

The compositions of NL and PL are reported in Table 1, in Section 2 of the manuscript. Both NL and PL extracts were dissolved and stored in ethanol (level of purity: ≥99.50%) at −20 °C under a nitrogen atmosphere and protected from light.

#### 4.2.3. HepG2 Proliferation Assay of Neutral and Polar Lipid Extracts of *P. tricornutum* Using MTT Method

Once the cells harvested, a Malassez cell count was carried out to calculate the volume to be taken according to the quantity of cells per well. Thus, 14,000 cells per well were deposited using a multichannel pipette at a rate of 100 μL. The plates were incubated for 24 h at 37 °C and 5% CO_2_. Then, the medium was removed and different concentrations of neutral and polar lipid extracts from *P. tricornutum* to be tested (25, 50, 75 μg/mL) were carried out in the weaning medium containing 1% BSA and incubated for 24 h at 37 °C and 5% CO_2_ before a test on viability to MTT. The 96-well plates were then incubated for 24 h at 37 °C and 5% CO_2_. The starving medium containing the different concentrations of lipid extracts was then gently removed and the MTT viability test performed according to the following process. The method is based on the use of tetrazolium salt MTT (3-(4,5-dimethylthiazol-2-yl)-2,5-diphenyl tetrazolium bromide). This salt contains a tetrazolium ring that is reduced to formazan by mitochondrial succinate dehydrogenase of metabolically active living cells. The reduction reaction forms purple-colored formazan crystals that will solubilize in the presence of DMSO. The precipitate content of formazan formed is then proportional to the amount of living cells present. Absorbance values are obtained using a microplate reader (Agilent Biotek, Les Ulis, France) at 540 nm wavelength to quantify living cells and then proliferation.

#### 4.2.4. HepG2 Cytotoxicity Assay of NL and PL Extracts of *P. tricornutum* Using the Lactate Dehydrogenase (LDH) Assay Kit

The LDH test was used to quantify cytotoxicity or cellular mortality. It has been based on measuring the activity of lactate deshydrogenase (LDH), which is a stable enzyme present in the cytoplasm of all cells and which is rapidly released into the cell culture supernatant after plasma membrane damage. These lesions may be caused by necrosis and/or cell lysis. LDH activity is determined by a coupled enzymatic reaction where LDH oxidizes lactate to pyruvate, which then reacts with IodoNitroTetrazolium (INT) to form red formazan. The amount of formazan produced in the culture supernatant is directly related to the number of cells lysed. Formazan being soluble in water can be detected using a microplate reader at 490 nm wavelength.

After treatment of HepG2 cells with NL and PL extracts from *P. tricornutum* at the different concentrations to be tested, i.e., 0, 25, 50, and 75 μg/mL, the plates were incubated for 24 h at 37 °C and 5% CO_2_. The starving medium with the different concentrations of lipid extracts was then gently removed and the LDH test was performed according to the manufacturer’s instructions (Roche Diagnostics, Hong Kong, China). Briefly, a lysis solution was added to the wells reserved for the high range control. A mixed solution was made from vial 1 (catalyst: enzyme diaphorase) and vial 2 (Dye solution: INT) of the enzyme kit. The mixture was distributed to each well, incubated for 3 min in the dark, and then the solution to stop the reaction was added to each well. The absorbance (A) reading was carried out with a microplate reader at 490 and 600 nm wavelength: the absorbance at 600 nm wavelength corresponding to the reaction with LDH and the one at 490 nm wavelength to the colored reaction forming formazan. The difference in absorbances obtained at these two wavelengths allowed the absorbance of formazan alone without interactions.

The percentage of cytotoxicity was calculated using the following formula:

cytotoxicity (%) = ((A assay − A low control)/(A high control − A low control)) × 100(1)

#### 4.2.5. Hoechst Staining on HepG2 Nuclei with or Without NL Extracts

For this study, 7000 cells/well in 200 μL of complete culture medium were introduced in 8-well slide (LabbioTek^®^, San Jose, CA, USA). The slides were incubated at 37 °C and 5% CO_2_ for 24 h. The culture medium was then removed and rinsed with 200 μL of PBS. A range of NL was performed with the following concentrations (0, 25, 50 and 75 μg/mL) in the weaning medium with 1% Bovine Serum Albumin (BSA). Then, each concentration of NL was added in the corresponding wells with a final volume of 200 μL. The slides were incubated at 37 °C and 5% CO_2_ for 24 h. Two hundred microliters of methanol at −20 °C were deposited in each well and the slides incubated 20 min at −20 °C. The methanol was removed and 100 μL of Hoechst reagent at 0.5 μg/mL added. Then, the slides were incubated for 20 min in the dark at room temperature. The Hoechst staining was removed, and the wells washed with 200 μL of ultrapure water. After discarding the ultrapure water, the wells covering the slides were removed. Slides were mounted with glycerol and lamella.

The observation of cells stained with Hoechst staining was performed using a fluorescence microscope with a ×100 objective with oil immersion. To check the presence of Hoechst reagent able to observe nuclei in blue, a 540 nm wavelength filter was used. The counting of the nuclei was carried out in duplicate on 10 fields per well. The labeling index (LI) can be described as a measure of the apoptotic activity of a cell population, defined as the number of cells in the apoptotic phase divided by the total cells in the population. Once the count was complete, LI was determined with the following formula:

LI (%) = (apoptotic nuclei/total nuclei) × 100 (2)

Microphotographs were taken to illustrate the apoptosis profile using the Lasez^®^ software version 3.4.0 coupled to a camera (Leica, Wetzlar, Germany).

#### 4.2.6. Palmitate-Treated HepG2 and Oil Red Staining with or Without NL and PL Fractions

HepG2 cells were seeded at a density of 150,000 cells/well in 6-well plates. HepG2 cells with NL or PL extracts from *P. tricornutum* were treated with different concentrations to be tested (0, 25, 50 and 75 μg/mL) in starving medium containing 5% BSA and 250 µg/mL palmitate. In addition, subsequent ethanol controls were made. Incubation was performed for 24 h at 37 °C and 5% CO_2_. The medium with the different concentrations of lipid extracts and controls ethanol was then gently removed and rinsed once with PBS. After 24 h of treatment, lipid droplets in HepG2 cells were revealed with an Oil Red staining kit from ScienCell Research Laboratories (Carlsbad, CA, USA), according to the manufacturer’s protocols. Briefly, cells were fixed, and 3 washes were performed. Oil Red was diluted at 20% in ultrapure water and filtered with a 0.22 µm filter. HepG2 LDs were stained with 1.5 mL of filtered Oil red for 20 min at room temperature followed by 5 ultrapure water washes. The images were acquired by optical microscopy using a magnification of 400. Then, the LDs previously stained with Oil Red were solubilized with 2 mL of isopropanol/well. The red coloration of the solution obtained was proportional to the LD accumulation in the HepG2 cells and was measured spectrophotometrically at a wavelength of 520 nm. Then, the subtraction of the different ethanol control with the lipid at a given concentration showed the effect on lipid droplet accumulation. Microphotographs were submitted to ImageJ software to determine the average size of the LD among the different conditions.

The inhibition rate (%) of lipid droplet formation was calculated using the following formula:

Inhibition rate (%) = ((A control − A assay (with NL or PL))/A control) × 100 (3)

### 4.3. Statistics

Results were obtained from triplicate or duplicate experiments when specified, and the data presented are the means +/− standard deviations (SDs) obtained after at least three independent experiments. The analysis of variance by one-way ANOVA followed by a Dunnett post hoc test was performed. Statistical analyses were carried out with Prism 9 (GraphPad Software, Boston, MA, USA), and the results were considered statistically different for values of *p* ≤ 0.05, *p* ≤ 0.01 or *p* ≤ 0.001 as indicated in the figure legends.

## 5. Conclusions

This preliminary study evidenced that neutral lipids from *P. tricornutum*, rich in PUFA n-3 (notably 20:5n-3), significantly reduced intracellular lipid droplet formation in palmitate-induced steatosis HepG2 cells, whereas polar lipids slightly increased lipid droplet size. Neutral lipids NL had no marked effect on cell viability at the effective concentration of 50 mg/mL, although reduced viability was observed at higher concentrations, likely through necrotic rather than apoptotic mechanisms. These results highlight NL as bioactive modulators of intracellular lipid metabolism and suggest their potential relevance for NAFLD prevention.

However, the palmitate-induced steatosis HepG2 model only partially reflects the complexity of NAFLD. Further studies using more physiologically relevant models as primary hepatocytes, liver organoids, and/or animal models are required to confirm these effects and elucidate the underlying mechanisms. Additional analyses, including assays to precisely characterize cell death mechanisms (e.g., Annexin V/PI staining and caspase activity) and also detailed lipidomic profiling to investigate intracellular lipid-class after treatment with NL and PL fractions would strengthen and extend these preliminary findings.

Overall, NL from *P. tricornutum* may be promising bioactive compounds for reducing hepatocyte lipid accumulation and provide a solid basis for future studies exploring their nutraceutical potential in NAFLD.

## Figures and Tables

**Table 1:** Fatty acid composition of neutral (NL) and polar (PL) lipid extracts (as average of 3 different lipid extractions) from *P. tricornutum*, in weight (mg of fatty acid/100 g microalga) and in % (of total fatty acids of NL and PL, respectively), ± standard deviation (sd).

Fatty Acids	Neutral Lipids			Polar Lipids		
	**mean**	**± sd**	**%**	**mean**	**± sd**	**%**
C14:0	94.167	69.954	4.15	40.333	29.670	4.87
C16:0	201.556	127.816	8.89	137.500	111.594	16.62
C16:1n-13tr?	61.944	36.781	2.73	nd	nd	nd
C16:1n-7	322.444	211.536	14.22	92.500	69.168	11.18
C16:2n-4	80.167	53.484	3.54	31.667	24.861	3.83
C16:3n-4	228.056	141.334	10.06	81.167	70.612	9.81
C18:0	27.222	14.849	1.20	18.500	23.468	2.24
C18:1n-9 (cis+trans)	30.056	17.113	1.33	21.000	18.520	2.54
C18:1n-7	nd	nd	nd	21.333	20.033	2.58
C18:2n-6cis	38.278	25.088	1.69	24.333	21.180	2.94
C20:4n-6	51.778	36.257	2.28	18.000	11.533	2.18
C20:4n-3	nd	nd	nd	10.000	9.179	1.21
C20:5n-3	922.611	626.803	40.69	179.333	94.028	21.67
C24:0	29.167	26.704	1.29	68.000	65.294	8.22
C22:6n-3	36.667	22.205	1.62	27.500	25.821	3.32
*SFA*	*382.278*	*258.897*	*16.86*	*285.500*	*249.474*	*34.50*
*MUFA*	*451.333*	*289.632*	*19.90*	*139.333*	*112.539*	*16.84*
*PUFA*	*1433.722*	*952.043*	*63.23*	*402.667*	*284.136*	*48.66*
*n-3 PUFA*	*1003.833*	*676.255*	*44.27*	*229.833*	*139.764*	*27.77*
*n-6 PUFA*	*113.000*	*76.151*	*4.98*	*58.333*	*47.933*	*7.05*
*n-4 PUFA*	*310.111*	*195.580*	*13.68*	*113.000*	*95.821*	*13.66*

SFA: saturated fatty acids; MUFA: monounsaturated fatty acids; PUFA: polyunsaturated fatty acids. The following fatty acids, in percentage < 1% of the total fatty acid methyl esters (FAME), were also found: iso-C14:0, C14:1n-7?, anteiso-C15:0, C15:0, iso-C16:0, C16:1n-9, anteiso-C17:0, C16:2n-6?, C17:0, C17:1?, C16:4n-1, C18:2n-4, C18:3n-6, C18:3n-3, C18:4n-3, C20:0, C20:1n-9, C20:2n-6, C20:3n-6, C20:3n-3, C22:0, C22:1n-9, C22:2n-6, C22:5n-3. They were utilized for calculating the sum of total fatty acids, but are not listed in the table. C16:1n-13tr?: not sure identification of this fatty acid. nd: not detected.

## Data Availability

The original contributions presented in this study are included in the article. Further inquiries can be directed to the corresponding author.
